# Transcriptomic Responses of a Lightly Calcified Echinoderm to Experimental Seawater Acidification and Warming during Early Development

**DOI:** 10.3390/biology12121520

**Published:** 2023-12-13

**Authors:** Ye Zhao, Mingshan Song, Zhenglin Yu, Lei Pang, Libin Zhang, Ioannis Karakassis, Panagiotis D. Dimitriou, Xiutang Yuan

**Affiliations:** 1 Key Laboratory of Coastal Zone Environmental Processes, Yantai Institute of Coastal Zone Research, Chinese Academy of Sciences, Yantai 264003, China; 2 Ocean School, Yantai University, Yantai 264005, China; 3 Ministry of Ecology and Environment, National Marine Environmental Monitoring Center, Dalian 116023, China; 4 CAS Key Laboratory of Marine Ecology and Environmental Sciences, Institute of Oceanology, Chinese Academy of Sciences, Qingdao 266071, China; 5 Marine Ecology Laboratory, Department of Biology, University of Crete, GR 70013 Heraklion, Greece

**Keywords:** climate change, muti-stressor, sea cucumber, *Apostichopus japonicus*, transcriptomics, early development

## Abstract

**Simple Summary:**

Marine organisms are facing multifaceted changes in their environment due to near-future ocean warming and acidification. We aimed to analyze the impacts of concurrent climate change stressors (seawater warming and acidification) on the embryo/larval development and gene expression patterns of the sea cucumber *Apostichopus japonicus*. Elevated temperatures have been found to expedite the embryo–larval development of *A. japonicus*, while decreased pH inhibited these processes, but the effect of combining the former two seemed to be slight. At the transcriptomic level, acidification induced attenuated biomineralization and skeletogenesis, and triggered the risk of extracellular acid–base imbalance and oxidative injury; warming introduced a noticeable stress response; while dual stresses strengthened these stress responses, resulting in reduced immune resistance and heightened system instability. This study provides new insights into the potential adaptive mechanisms employed by sea cucumbers and projects the potential effects of climate change on marine organisms and ecosystems.

**Abstract:**

Ocean acidification (OA) and ocean warming (OW) are potential obstacles to the survival and growth of marine organisms, particularly those that rely on calcification. This study investigated the single and joint effects of OA and OW on sea cucumber *Apostichopus japonicus* larvae raised under combinations of two temperatures (19 °C or 22 °C) and two *p*CO_2_ levels (400 or 1000 μatm) that reflect the current and end-of-21st-century projected ocean scenarios. The investigation focused on assessing larval development and identifying differences in gene expression patterns at four crucial embryo–larval stages (blastula, gastrula, auricularia, and doliolaria) of sea cucumbers, using RNA-seq. Results showed the detrimental effect of OA on the early development and body growth of *A. japonicus* larvae and a reduction in the expression of genes associated with biomineralization, skeletogenesis, and ion homeostasis. This effect was particularly pronounced during the doliolaria stage, indicating the presence of bottlenecks in larval development at this transition phase between the larval and megalopa stages in response to OA. OW accelerated the larval development across four stages of *A. japonicus*, especially at the blastula and doliolaria stages, but resulted in a widespread upregulation of genes related to heat shock proteins, antioxidant defense, and immune response. Significantly, the negative effects of elevated *p*CO_2_ on the developmental process of larvae appeared to be mitigated when accompanied by increased temperatures at the expense of reduced immune resilience and increased system fragility. These findings suggest that alterations in gene expression within the larvae of *A. japonicus* provide a mechanism to adapt to stressors arising from a rapidly changing oceanic environment.

## 1. Introduction

CO_2_ emissions resulting from human activities since the Industrial Revolution have caused global climate change. The substantial release of CO_2_ and other greenhouse gases and the excessive absorption of CO_2_ by the ocean can induce ocean acidification (OA) and ocean warming (OW) [1,2]. OA and OW pose a huge threat to various marine organisms, particularly those with calcified structures. Ocean pH has been projected to decline by 0.3–0.4 units and temperature to increase by 1.5 °C–3 °C by the end of the 21st century [3]. OA is accompanied by a decrease in the saturation of calcium carbonate minerals required to form skeletons and the occurrence of hypercapnia [4]. OW has a promotional effect on physiological processes, whereas hypercapnia induced by OA has a suppressive effect [5,6]. Additionally, the combination of OA and OW may have unpredictable deleterious and/or beneficial interactive effects on marine biota. Therefore, investigating the interactive effects of OA and OW when assessing risks to marine biota is a critical concern.

The impact of high temperature or high *p*CO_2_ as a sole stressor on marine life has been widely studied. In particular, research regarding OA has aroused broad concern. Investigations on the impacts of climate change on embryo/larvae histories have largely focused on OA as the sole stressor [7,8]. A single-stressor investigation of OA showed impaired development in echinoderm larvae reared in acidified conditions projected for the year 2100 [9,10]. Many studies have attempted to understand the response of echinoderms to OA from a molecular perspective in recent years, especially through transcriptomic analysis. Studies that investigated the effects of low pH on gene expression in sea urchin larvae indicated that OA significantly altered the expression of genes related to cell division, metabolism, biomineralization, and antioxidation [11,12,13]. A study of sea urchin larvae reared in pH 7.6 conditions suggested an increase in genes related to immune activity as a consequence of pathogen resistance and animal survival [14]. A single study of high seawater temperature suggested that temperature affected early development, survival, growth, metabolism, immunity, and gene expression profile in echinoderms [15,16,17]. Juvenile sea urchin *Loxechinus albus* can respond to OW via regulating differential genes related to heat shock, membrane potential, and detoxification [18]. Given that OW and OA will happen simultaneously under future climate change conditions, the combined effects of these multiple global change drivers on marine biota are receiving increasing attention [19,20,21,22]. The majority of these studies reported the effects of OW and OA on the early stages of commercially important species, since the impacts of stressors can be devastating to early life stages and can increase mortality in these stages, leading to serious impacts on population trends. For example, a study on sea urchin embryo/larvae development suggested that acidification and warming may have an additive inhibition on fertilization rates and larval morphological development [23]. Moreover, warming can allow starfish to better cope with the adverse effect of acidification on calcification [24].

Most studies involved in the responses of echinoderms to climate change have focused on heavily calcified sea urchin echinoderms, whereas lightly calcified echinoderms, such as sea cucumber, have been given less attention [25]. The sea cucumber *Apostichopus japonicus* is an essential economic and ecological marine species, with habitats ranging from 0 to 50 m [26,27]. The gonads of *A. japonicus* mature from June to August, and the temperature required for it to spawn ranges from 19 °C to 20 °C [26]. This species plays a crucial role in structuring coastal ecosystems and facilitating nutrient exchange and the carbonate cycle at the water–sediment interface, acting as an “ecosystem engineer” [28,29]. Here, the interactive effect of OA and OW on the larvae of *A. japonicus* was investigated, focusing on the embryo/larval stage, because this life stage is highly susceptible to seawater chemistry and temperature [26,27]. Transcriptomics, as the latest developed technology, has played an essential role in biological research. The basic methods of transcriptome research include microarray technology, the serial analysis of gene expression (SAGE), massively parallel signature sequencing (MPSS), expressed sequence tags (EST), and high-throughput sequencing. Comprehensive analysis at the transcriptomic level using RNA-seq has been shown to reflect the physiological status of marine organisms [30,31]. In sea cucumbers, RNA sequencing has been used to understand the underlying mechanisms of skin ulceration syndrome, regeneration, and aestivation [32,33,34]. Meanwhile, transcriptomic analysis preliminarily elucidates the immune responses of sea cucumbers to seasonal high temperatures [35]. However, little transcriptomic information is known regarding the responses of sea cucumbers to climate change [36].

This study investigated the single and joint effects of OA and OW on the embryos and larvae of sea cucumbers raised under combinations of two temperatures (19 °C vs. 22 °C) and two *p*CO_2_ levels (400 μatm vs. 1000 μatm), covering the present situation and scenarios of OA and OW by the end of the 21st century. In this investigation, the development of embryo/larvae was assessed, and differences in gene expression patterns under OA and OW were identified. This study aimed to provide insights into the potential adaptive mechanisms employed by sea cucumbers and project the potential effects of climate change on marine organisms and ecosystems by analyzing these molecular-level responses.

## 2. Materials and Methods

### 2.1. Experimental Seawater Manipulation

The seawater used in this experiment was pumped from the Dalian coast and filtered using a composite sand filter into a large reservoir tank. The seawater acidification and warming system was manipulated in four artificial climate incubators (HP400G-D, Wuhan Ruihua Instrument and Equipment, Wuhan, China) (Figure S1). Pure CO_2_ gas (99.9%) was pumped into the CO_2_ enrichment incubators and mixed with air to achieve the desired atmospheric concentrations (400 and 1000 μatm). The CO_2_ concentration and temperature were automatically regulated using a CO_2_ detector VC1008F and a temperature conductor, respectively, to cover the present and future scenarios of OA and OW by the end of the 21st century. Four experimental treatments were set up: 19 °C, 400 μatm (CON); 19 °C, 1000 μatm (OA); 22 °C, 400 μatm (OW); and 22 °C, 1000 μatm (OAW). Among them, CON was regarded as the control group, and OAW was used to simulate the ocean environment by the end of the 21st century. Four glass aquariums (40 cm × 40 cm × 20 cm) were included in each incubator and used for culturing embryos and larvae, which allowed for four treatment groups to be tested in four replicates in each group.

Seawater pH was monitored daily using an S20P-K pH meter with a sensitivity of 0.001 units (Mettler Toledo Instrument, Shanghai, China) and calibrated with NBS buffers. The seawater salinity and temperature were monitored daily using a YSI 6600V2 handheld multiparameter instrument (YSI Incorporated, Yellow Springs, OH, USA). Seawater total alkalinity (AT) was measured weekly during the experiment using a Total Alkalinity Gran Titration System (AS-ALK2, Apollo SciTech, Bogart, GA, USA). Calcite and aragonite saturation states and *p*CO_2_ values were calculated using CO2SYS software (v16) [37].

### 2.2. Artificial Fertilization and Animal Culture

Fifty mature adult *A. japonicus* (body length 18–22 cm, body weight > 200 g) were collected from the Dalian coast, transported to the laboratory, and cultured temporarily in five tanks (1.5 m^3^). Fertilization was conducted by the emission of sperm and eggs from male and female individuals, followed by the mixing of sperm and egg in a ratio of 10 sperm to 1 egg to prevent polysemy. The fertilized eggs were then divided into 16 glass aquariums within 4 incubators for early development, with a final concentration of 1 egg per mL. Once the larvae reached the early auricularia stage (~43 h post-fertilization (hpf)), they were fed two times a day with the oceanic red microalga *Rhodotorula* sp. at a cell density of 1.5 × 10^6^ cell mL^−1^. Prior to reaching the early auricularia stage, seawater changes were performed by adding water, and then the seawater was changed by 1/2 per day.

### 2.3. Sample Collection

Considering the impact of experimental stressors on developmental asynchrony, the embryos and larvae were selected for sampling at the target phases, ensuring that over 80% of individuals reached these phases (for blastula 8 hpf; for gastrula 19 hpf; for late-auricularia 3 d post-fertilization (dpf); for doliolaria 10 dpf), as confirmed by microscopy (Nikon YS100, Nikon, Tokyo, Japan). The development duration was calculated by recording the developmental time (accurate to a minute). A total of 50 larvae at four crucial developmental stages (blastula, gastrula, late-auricularia, and doliolaria) were randomly collected from each of the four aquariums within the incubator to assess the developmental state. These samples were then transferred into a sampling bottle (50 mL) for the observation of the embryo/larvae development state and the measurement of larval anterior–posterior body length under a microscope using an ocular micrometer. In addition, 0.1 g sea cucumber embryos and larvae (blastula, gastrula, auricularia, and doliolaria) were collected and frozen immediately with liquid nitrogen for further transcriptome analysis.

The growth rate of larvae at four planktonic stages was calculated using the following formula:

Growth rate = (Lt − Li)/t,

where Li represents the initial body length, Lt represents the terminal body length, and t denotes the development time in days.

### 2.4. RNA Extraction, Library Construction, and Transcriptomic Sequencing

Total RNA was extracted using the TRIzol reagent following the manufacturer’s instructions, and its quantity and quality were measured using Nanodrop, Qubit, and gel electrophoresis. The completeness of total RNA was measured using Agilent 2100 (Agilent, Santa Clara, CA, USA). The mRNA was subjected to further enrichment using magnetic beads with oligo (DT), and fragment buffer was introduced to facilitate the fragmentation of mRNA into shorter segments. One strand of complementary DNA (cDNA) was synthesized utilizing mRNA as a template, employing six base random hexamers. Subsequently, two strands of cDNA were synthesized by incorporating buffer, dNTPs, DNA polymerase I, and RNase H. Following purification using AMpure XP beads, the cDNA was subjected to repair, and the poly tail and connected sequencing adaptor ligation were added. After the cDNA fragment size was selected and PCR amplification was performed, the PCR products were purified by AMpure XP beads to obtain libraries. Finally, the libraries were sequenced using Illumina Hiseq by Novogene Co., Ltd. (Beijing, China).

### 2.5. Sequence Assembly

Raw reads were filtered by eliminating low-quality reads and adapters to obtain clean reads. The clean reads were subjected to assembly using the program Trinity [38]. This program efficiently handles a substantial volume of clean reads through the integration of three distinct software modules: Inchworm, Chrysalis, and Butterfly. Inchworm effectively disassembles reads to generate contigs, while Chrysalis clusters overlapping contigs to form components. Each component represents a collection of groups that consist of alternative isoforms or potential characterizations of homologous genes, each accompanied by its corresponding de Bruijin graph. Butterfly simplifies the de Bruijin graph of each component, producing full-length transcripts of alternative isoforms and ultimately yielding the desired assembly results. The longest transcript is selected as the representative gene.

### 2.6. Identification of Differentially Expressed Genes (DEGs)

The assembled transcriptome using Trinity was considered as the reference sequence (ref), and the clean reads from each sample were mapped against the reference by using the RSEM software (v0.6) to obtain the read count [39]. The number of clean tags exclusively mapped to each gene was quantified and normalized using the reads-per-kilobase-million-reads method to analyze the gene expression. In this study, the DEGs were identified by setting a *p*-value of 0.05 and an absolute value of log_2_Ratio ≥ 1. The composition structure of gene expression profiles could be visualized on a two-dimensional map using Principal Coordinates Analysis (PCoA) based on the Bray–Curtis distance. Origin software (v8.0) could be used to plot the overall differences and differences in the different treatment and developmental stages.

The main functional classifications of DEGs can be recognized via Gene Ontology (GO) and Kyoto Encyclopedia of Genes and Genomes (KEGG) analyses. The DEGs were mapped to terms using the GO database (http://www.geneontology.org/ (accessed on 30 September 2019)) and the KEGG database (http://www.genome.jp/kegg/ (accessed on 30 September 2019)), and a hypergeometric test with a false discovery rate (FDR) of ≤0.05 was employed to determine the significant enrichment of GO terms and pathways in the DEGs.

### 2.7. Quantitative PCR

The transcriptome results were further validated by measuring the mRNA expression of genes related to biomineralization, immune system, and energy metabolism in the same larval samples from the OA, OW, and OAW treatments by using quantitative real-time PCR (qPCR). The total RNA was isolated using the TaKaRa MiniBEST Universal RNA Extraction Kit (TaKaRa Code. 9769), in accordance with the manufacturer’s instructions. The quality and quantity of RNA were measured using the Nano Photometer and agarose gel electrophoresis. RNA was used to synthesize cDNA by using the PrimeScript RT reagent Kit with a gDNA Eraser (TaKaRa Code. RR047). The cytochrome b (cy*tb*) gene was used as the reference. The qPCR reaction was performed in a total volume of 25 µL containing 12.5 µL of TB Green Premix Ex Taq II (2×, TB Green Premix Ex Taq II (Tli RNaseH Plus), TaKaRa Code. RR820), 2 µL of cDNA template, 8.5 μL of RNase free water, and 1 µL of each primer (primer sequences can be found in Table S1). The qRT-PCR program was performed at 95 °C for 30 s, followed by 40 cycles of 95 °C for 5 s, at annealing temperature for 20 s, and at 72 °C for 20 s. The relative mRNA levels of the target genes were calculated via the 2^−ΔΔ*Ct*^ method.

### 2.8. Statistical Analysis

All statistics were evaluated using SPSS software (version 22, IBM Corp., Armonk, NY, USA). The data normality and homogeneity of variance were checked. In accordance with data normality, a nonparametric Kruskal–Wallis test or ANOVA was chosen. Duncan’s test or Dunnett’s T3 test was chosen for the one-way ANOVA based on the homogeneity of variance. Data were presented as mean ± SD, and differences were accepted as statistically significant at *p* < 0.05.

## 3. Results

### 3.1. Seawater Carbonate Chemistry

Elevated *p*CO_2_ and/or increased temperature exerted no significant effect on water salinity (*p* = 0.99) and TA (*p* = 0.99), but they significantly affected all other parameters of the carbonate system: pH, temperature, *p*CO_2_, Ω_Ca_, and Ω_Ar_ (all *p* < 0.05). As shown in Table 1, the seawater pH_NBS_ values were 8.08, 7.74, 8.12, and 7.72, and the temperatures were 19.1 °C, 19.0 °C, 22.3 °C, and 22.1 °C in CON (19 °C, ~400 μatm), OA (19 °C, 1000 μatm), OW (22 °C, ~400 μatm), and OWA (22 °C, 1000 μatm) groups, respectively. The pH values of the experimental seawater with elevated *p*CO_2_ decreased by 0.34–0.40 units (an average of 0.37 units), and the water temperature increased by 3.0 °C–3.3 °C (an average of 3.1 °C) compared with those of the ambient control.

### 3.2. Effect of Seawater Acidification and Warming on Early Development

**Figure 1:**
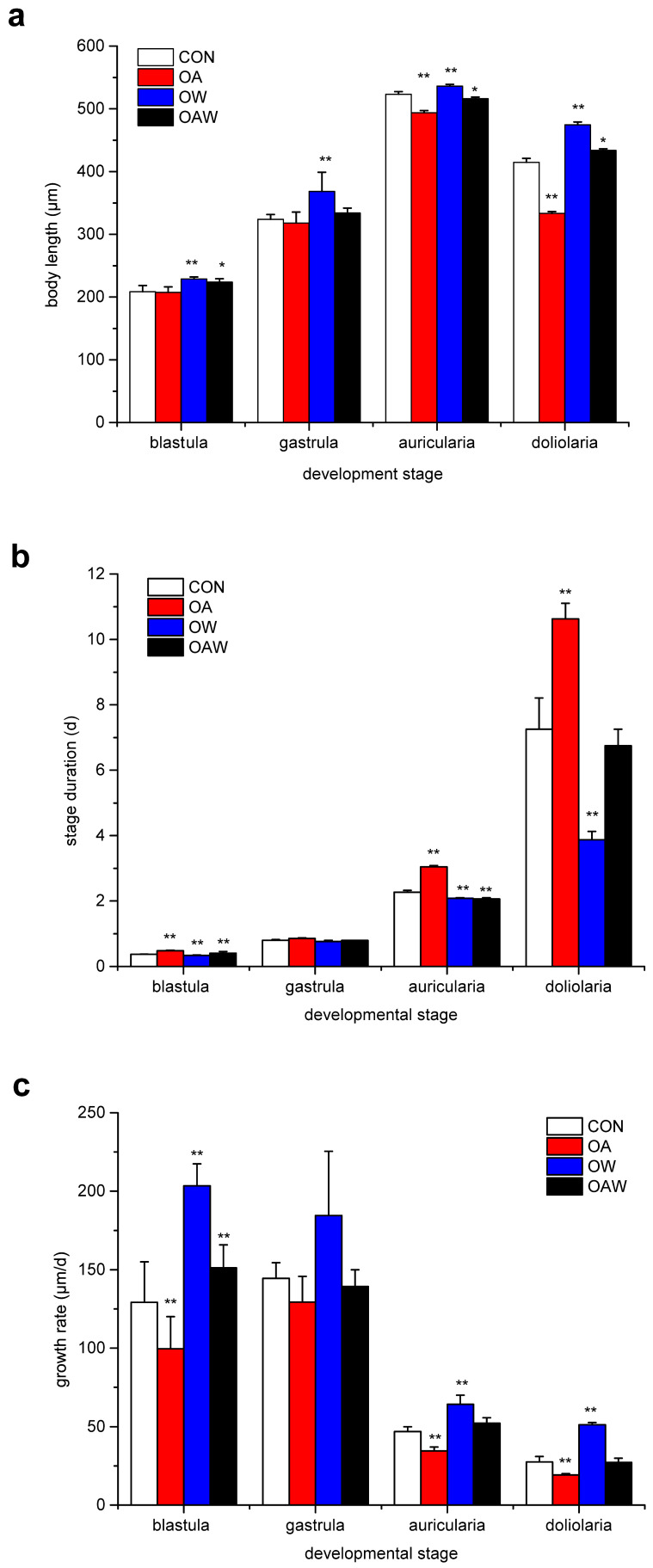
Effect of OA and/or OW on larval development and growth of larvae of *A. japonicus.* (**a**) Body length of *A. japonicus* larvae (blastula: *n* = 4, LSD, F_3,12_ = 8.724, *p* = 0.019; gastrula: *n* = 4, LSD, F_3,12_ = 6.017, *p* = 0.01; auricularia: *n* = 4, LSD, F_3,12_ = 113.820, *p* < 0.0001; doliolaria: *n* = 4, LSD, F_3,12_ = 800.676, *p* < 0.0001). (**b**) Stage duration of *A. japonicus* larvae (blastula: *n* = 4, LSD, F_3,12_ = 114.723, *p* = 0.525; gastrula: *n* = 4, LSD, F_3,12_ = 9.333, *p* = 0.006; auricularia: *n* = 4, LSD, F_3,12_ = 521.214, *p* = 0.002; doliolaria: *n* = 4, LSD, F_3,12_ = 84.000, *p* < 0.0001). (**c**) Growth rate of *A. japonicus* larvae (blastula: *n* = 4, LSD, F_3,12_ = 20.506, *p* = 0.115; gastrula: *n* = 4, LSD, F_3,12_ = 4.370, *p* = 0.027; auricularia: *n* = 4, LSD, F_3,12_ = 21.516, *p* < 0.0001; doliolaria: *n* = 4, LSD, F_3,12_ = 132.188, *p* < 0.0001). OA: ocean acidification; OW: ocean warming. The “**” symbol above the bar chart indicates a significant difference (*p* < 0.01), and the “*” symbol above the bar chart indicates a significant difference (*p* < 0.05).

This study demonstrates that OA and/or OW exert a notable influence on the embryo/larvae development of *A. japonicus*. As seen in Figure 1, OA resulted in a significant reduction in body length during the later stages of *A. japonicus* (auricularia and doliolaria stages, *p* < 0.01) compared with the CON treatment. Conversely, the OW treatment led to a significant increase in body length compared with the CON treatment across all four larval stages (*p* < 0.01). Furthermore, the combined treatment of OAW significantly promoted body growth during the blastula, auricularia, and doliolaria stages (*p* < 0.05). Similar patterns were observed in terms of growth-stage duration and growth rate (Figure 1b,c), with OA significantly prolonging the development time and OW significantly shortening it compared with that in the CON group (*p* < 0.05). In addition, the growth rate was significantly suppressed and accelerated in the OA and OW groups, respectively, at the blastula, auricularia, and doliolaria stages (*p* < 0.05).

### 3.3. Transcriptomic Responses to OA and OW

#### 3.3.1. Summary of Statistics and Overview of RNA-Seq

A total of 16 RNA-seq libraries (treatments of CON, OA, OW, and OAW at four developmental stages) were generated to comprehensively characterize the transcriptional profile of *A. japonicus* during early developmental stages under varying temperature and *p*CO_2_ conditions, resulting in 2,727,597,920 raw reads (Table S2). Following the removal of useless reads (adaptor reads, 10% unknown bases, and low-quality reads), an average of 55,567,626 clean reads per library were retained, representing an average retention rate of 97.79% of the raw reads. Moreover, an average of 76.68% clean reads were successfully aligned to the reference genome of *A. japonicus*, with Q20 percentages exceeding 98% and Q30 percentages surpassing 94%. The sequence data from the present study were submitted to the NCBI Sequence Read Archive (http://www.ncbi.nlm.nih.gov/sra (accessed on 1 December 2023)) under the accession number PRJNA1019474.

#### 3.3.2. Distinct Transcriptomic Patterns during Early Development

**Figure 2:**
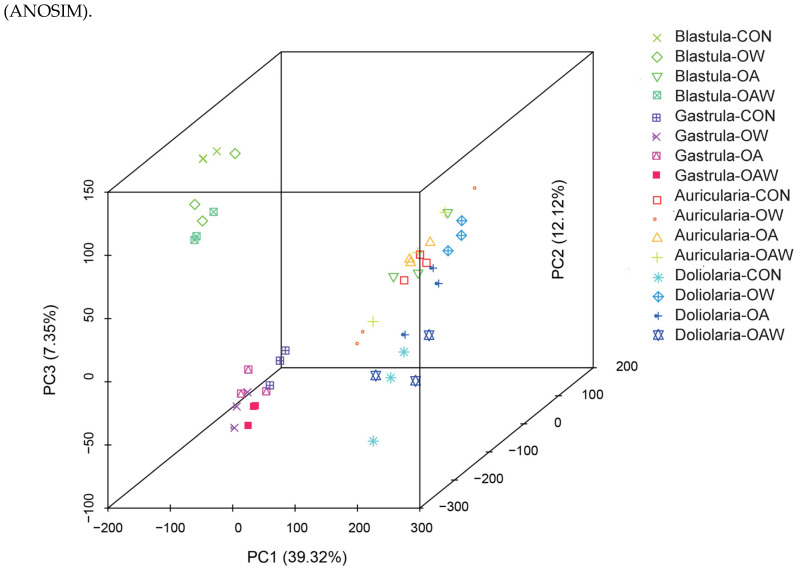
Principal component analysis (PCoA) of samples in four treatments at four larval stages of *A. japonicus* based on the Bray–Curtis distance.

Principal component analysis (PCA) of sample-to-sample distances was conducted to investigate the difference in gene expression profiles of sea cucumbers exposed to different combinations of temperature and *p*CO_2_ during early development (Figure 2). Principal component (PC) 1 represented the majority of the variance (39.32%), whereas PC2 and PC3 captured 12.12% and 7.35% of the variance, respectively. A clear separation between blastula- and gastrula-stage embryos could be observed, whereas those at the auricularia and doliolaria stages seemed not to be completely in accordance with the developmental stage. A notable detail was that the CON and OW groups at the auricularia stage, the OW group at the blastula stage, and the OA group at the doliolaria stage revealed closer clustering than other groups. These findings suggest that disparities in gene expression profiles during the gastrula stage are primarily influenced by the developmental stage, whereas the other three stages are influenced by the developmental stage and environmental stresses.

#### 3.3.3. DEG Analysis

**Figure 3:**
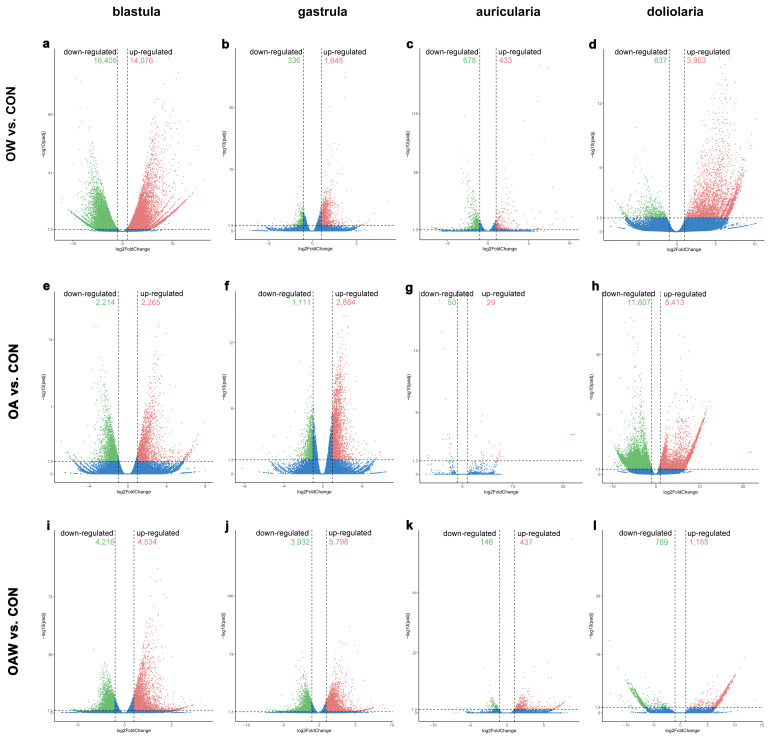
Volcano plots of DEGs for comparison of OA vs. CON (**a**–**d**), OW vs. CON (**e**–**h**), and OWA vs. CON (**i**–**l**) at four larval stages of *A. japonicus.* The principle “a *p*-value ≤ 0.05 and the absolute value of log_2_Ratio ≥ 1” was used as a threshold to screen DEGs. DEGs: differentially expressed genes; OWA: ocean warming and acidification; CON: control group.

The transcriptome results provided a global gene expression profile of *A. japonicus* under different combinations of elevated temperature and *p*CO_2_ during four early developmental stages. As seen in Figure 3, 87,896 DEGs were finally identified, including 28,773, 38,116, and 21,007 DEGs for three comparisons (OA vs. CON, OW vs. CON, and OAW vs. CON, respectively). Specifically, the most abundant DEGs appeared in two comparisons of OW vs. CON at the blastula stage (14,076 upregulated DEGs (16.73%, 14,706/87,896) and 16,408 downregulated DEGs (18.67%, 16,408/87,896)) and OA vs. CON at the doliolaria stage (8413 upregulated DEGs (9.57%, 8413/87,896) and 11,807 downregulated DEGs (13.43%, 11,807/878,96)). However, few DEGs among the three comparisons (2.02%, 1773/87,896) were detected at the auricularia stage. Of these DEGs, 2166, 1376, 14, and 99 DEGs were shared among the three comparisons at the blastula, gastrula, auricularia, and doliolaria stages, respectively (Figure S2), and the most abundant unique DEGs existed in the comparison of OW with CON at the blastula stage (25,049 unique DEGs (28.50%, 25,049/87,896) and OA vs. CON at the doliolaria stage (18,665 unique DEGs (21.24%, 18,665/87,896)).

In accordance with the primary findings, a selection of key DEGs that play significant roles in responding to environmental stress caused by OA and OW are emphasized and documented in Table 2. These key DEGs are categorized into four distinct groups: the development and regulation of the cell cycle, immune response and antioxidant defense, biomineralization and osteoblast, and the transport of calcium ions and maintenance of ion homeostasis. A notable detail is that biomineralization- and osteoblast-related DEGs, such as *Alx1*, fibroblast growth factor receptor, vascular endothelial growth factor receptor, C-type lectin, carbonic anhydrase, vacuolar protein sorting-associated protein, and *perlucin 5*, were downregulated at the doliolaria stages when exposed to OA treatment. In addition, immune- and antioxidant-defense-related DEGs, such as complement factor, cathepsin, heat shock protein, superoxide dismutase, glutathione S-transferase, scavenger receptor cysteine-rich domain superfamily protein, and glutathione peroxidase, were widely upregulated in OAW at different developmental stages of *A. japonicus*.

#### 3.3.4. GO and KEGG Classification

**Figure 4:**
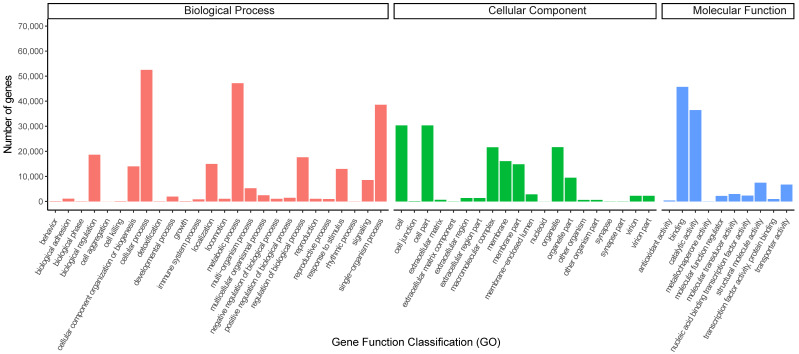
Histogram of GO pathways enriched by DEGs. The gene numbers enriched in GO terms of the categories “Biological Process”, “Cellular component” and “Molecular function” are shown. GO: gene ontology.

GO analysis was applied in accordance with an international standardized gene functional classification system to gain an in-depth comprehension of the functional classification of annotated genes. A total of 93,098 annotated genes were assigned to GO classes, including 242,854, 156,421, and 105,203 annotated genes assigned to biological process (BP), cellular component (CC), and molecular function (MF) GO terms, respectively, and 56 subcategory functional terms (GO level 2, Figure 4). In BP, the three largest subcategories were the cellular process (52,496), metabolic process (47,163), and single-organism process (38,591). Under CC, cell part (30,328), cell (30,328), organelle (21,655), and macromolecular complex (21,624) were prominently represented. For MF, binding (45,715) and catalytic activity (36,457) were the major subcategories among the annotated genes.

**Figure 5:**
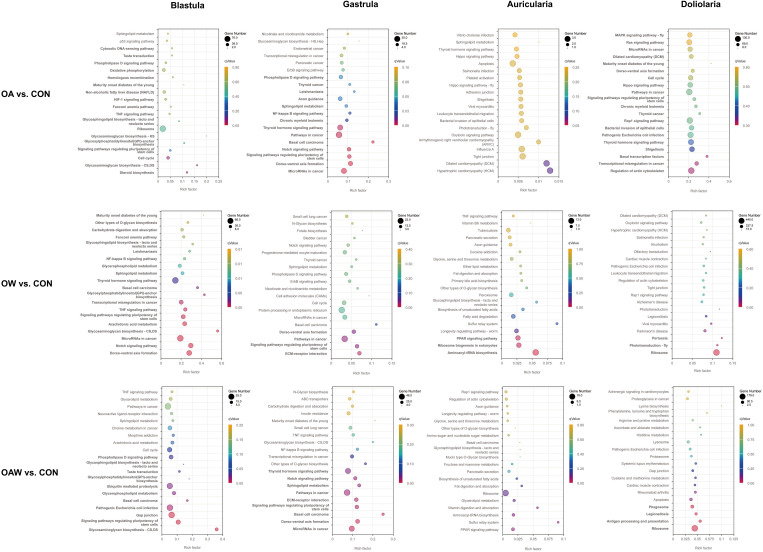
Bubble chart of top 20 KEGG pathways enriched by DEGs. Significantly enriched KEGG pathways are marked in bold and identified with q Value ≤ 0.05. KEGG: Kyoto Encyclopedia of Genes and Genomes.

An enriched pathway analysis of DEGs was performed to further understand the biochemical pathways involved in OA and OW. The top 20 KEGG enrichment results in each comparison at each developmental stage are displayed in Figure 5. All the top 20 KEGG pathways were significantly enriched in OW vs. CON at the blastula stage and OA vs. CON at the doliolaria stage (*p* < 0.05). The representative KEGG pathways included dorso–ventral axis formation (ko04320), notch signaling pathway (ko04330), and microRNAs in cancer (ko05206) in OW vs. CON at the blastula stage, and the regulation of the actin cytoskeleton (ko04810), transcriptional misregulation in cancer (ko05202), and basal transcription factors (ko03022) in OA vs. CON.

#### 3.3.5. Validation of DEG Analyses

**Figure 6:**
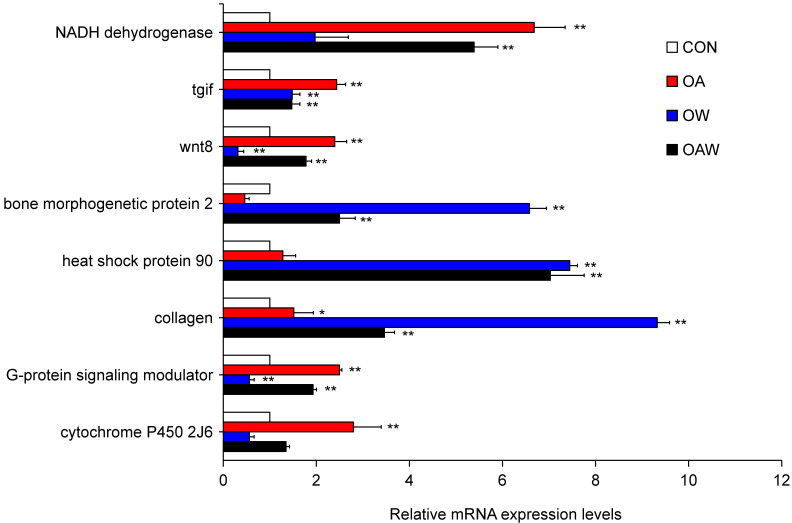
qRT-PCR results of eight DEGs in *A. japonicus* larvae under OA and/or OW. Data are represented as mean ± SD. The “**” symbol above the bar chart indicates a significant difference (*p* < 0.01), and the “*” symbol above the bar chart indicates a significant difference (*p* < 0.05). qRT-PCR: quantitative real-time polymerase chain reaction.

A total of eight genes (*NADH dehydrogenase*, *tgif*, *wnt8*, *bone morphogenetic protein 2*, *heat shock protein 90*, *collagen*, *G-protein signaling modulator*, and *cytochrome P450 2J6*) were chosen to assess their relative mRNA expression levels through RT-qPCR to authenticate the identified DEGs. The findings demonstrated that these eight genes displayed analogous patterns in terms of relative gene expression levels, as observed through RT-qPCR, with the DEG analysis patterns obtained from RNA-seq (Figure 6), thereby suggesting the reliability of the sequencing data.

## 4. Discussion

Seawater acidification and warming affect the survival and growth of many marine species, especially during critical life stages for the recruitment and replenishment of populations, such as embryo/larval stages [7,8]. It is worth noting that larval calcifiers are considered remarkably vulnerable to OA. For example, studies have demonstrated delayed early development and impaired body growth of heavily calcified echinoderms exposed to elevated *p*CO_2_, such as sea urchins and sea stars [9,10,23]. In the present study, the potential impacts of OA and OW on a lightly calcified echinoderm (i.e., sea cucumbers) were explored at the epigenetic developmental and transcriptomic levels.

The early larval development of *A. japonicus* was significantly influenced by OA and OW, with variations in sensitivity observed among different larval stages, leading to contrasting responses to OA and OW. Elevated temperatures have been found to expedite the larval development of *A. japonicus* across four stages. Notably, a temperature increase of +3.1 °C resulted in a 57.42% enhancement in larval growth rate during the blastula stage and a 46.55% reduction in the growth phase duration during the doliolaria stage. However, previous investigations on sea urchins revealed that elevated metabolic rates can restrict growth potential [40], thus explaining the modest increase observed in body length under OW conditions compared with that in control individuals. Furthermore, the present study reveals an inhibitory effect of embryo/larval development in *A. japonicus* under OA, especially pronounced during the doliolaria stage, as acidification led to a significant decrease of 19.65% in larval body length compared with the control group. This finding suggests that a complex and stage-specific response to OA may be considered in the early development of *A. japonicus* [28]. As pointed out by Byrne et al. [41] and Wolfe et al. [42], subsequent metamorphic stages in sea urchins exhibit greater susceptibility to pH changes than earlier stages, as evidenced by survivorship rates and skeletal morphology. In the case of *A. japonicus*, the doliolaria stage represents a crucial metamorphosis phase that undergoes a significant ecological transition from planktonic to benthic habitats, likely involving the initiation of internal skeleton formation [43]. Increased larval-stage duration of sea cucumber larvae under OA is likely due to higher metabolic cost compared to control. Previous studies hypothesized that a high CO_2_ scenario leads to increased metabolic costs due to hypercapnia and the consequent acid–base regulation, which could compromise the energy available for growth and other physiological functions of the fish [44]. The joint effect of elevated temperature and *p*CO_2_ values on the early larval development of sea cucumbers seems to be slight, suggesting that the detrimental effects of elevated *p*CO_2_ were counterbalanced by the expedited development under increased temperature. However, a different common effect of warming and acidification has been demonstrated in the case of other marine larvae, such as the green sea urchin *Lytechinus variegatus*, resulting in reduced fertilization rates, accelerated larval development, and the production of smaller and more asymmetrical larvae [23].

### 4.1. Transcriptomic Responses to OA and OW across Early Stages

In general, the molecular response of organisms to environmental stress manifests itself earlier and with greater sensitivity than physiological and phenotypic changes. This phenomenon highlights the potential of molecular processes, such as gene expression levels, in elucidating the underlying mechanisms of physiological and phenotypic plasticity under stress conditions. The utilization of RNA-seq analyses, which enable the simultaneous measurement of gene expression for all genes, offers a robust and unbiased approach to comprehensively understanding the adaptation mechanisms of sea cucumbers in response to OW and OA.

#### 4.1.1. Molecular Mechanisms Underpinning Sea Cucumber’s Resilience to OA

In this study, significant variations were observed in the number of DEGs identified across four developmental stages of sea cucumbers exposed to OA stress. These findings suggest the presence of stage-specific gene expression profiles that contribute to the resilience of sea cucumbers to OA during the early larval cycle. Notably, the doliolaria stage exhibited the highest number of DEGs, predominantly downregulated, which aligns with the previously reported delayed development observed in the study. These results imply that elevated *p*CO_2_ levels may have particularly detrimental effects on the metamorphosis stage of sea cucumbers. Indeed, unique molecular responses to OA during the metamorphosis stage were observed in metamorphosing juvenile sea urchins (*Heliocidaris erythrogramma*) [13]. As previously mentioned, the doliolaria stage represents the early phase of metamorphosis in sea cucumbers, involving the intricate coordination of developmental processes with increased energy expenditure, which may make this stage particularly susceptible to stressors [45]. This elevated vulnerability to stressors may explain the extensive downregulation of transcript levels, potentially indicating adaptive trade-offs in energy allocation to enhance resilience against OA.

A set of OA-responsive DEGs in *A. japonicus* was further highlighted to explore the molecular mechanisms involved in resilience to OA. These DEGs were found to align with previous research, particularly in the areas of stress response, biomineralization, and cellular homeostasis regulation. A cluster of genes associated with molecular protection and antioxidant defenses notably exhibited upregulation, indicating that exposure to low pH levels induced oxidative stress and perturbed intracellular redox homeostasis. Several previous studies provided evidence of the correlation between oxidative stress resulting from exposure to CO_2_ and increased levels of antioxidant proteins or enzyme activity [46,47]. One potential explanation for this phenomenon is that the reduction in internal pH likely enhances anaerobic respiration, leading to an increase in the production of reactive oxygen species (ROS), and the interaction between ROS and CO_2_ further amplifies this tendency [46].

Another noteworthy discovery is the significant downregulation of a set of crucial genes involved in the biomineralization process under elevated *p*CO_2_, specifically during the doliolaria stage. Biomineralization is a critical process of skeleton formation in various marine calcifying organisms, serving purposes such as defense, feeding, motility, and other important functions [48]. In the case of echinoderms, the development of skeletons likely occurred independently within each metazoan phylum [49]. Unlike their heavily calcified echinoderm counterparts, such as sea urchins with a well-developed endoskeleton, sea cucumbers possess only internal microscopic skeletal ossicles embedded within the dermal connective tissue of their body wall. This discrepancy can be attributed to the contracted biomineralization genes in the genome of *A. japonicus* [50]. So far, the developmental stage at which ossicles form during metamorphosis in *A. japonicus* larva, and the cellular and molecular mechanisms involved in this process, remain unclear. Qiu et al. [43] observed the initial presence of ossicles in the body wall during the early pentactula stage following the doliolaria stage. Therefore, it can be speculated that the doliolaria stage is when sea cucumbers initiate the formation of their internal skeleton. This speculation aligns with the doliolaria-stage-specific transcriptomic response to OA observed in this study, which involved genes relevant to biomineralization. The decrease in carbonate ion concentration caused by low pH environments can significantly impede the calcification rate at which marine organisms form skeletons and shells. Numerous studies examining gene expression in mollusks, corals, and urchins have indicated a clear and direct correlation between the downregulation of biomineralization- and osteoblast-related transcripts and exposure to low pH [49,51,52].

Among these genes, carbonic anhydrase (CA) is a key enzyme that converts CO_2_ to HCO_3_^−^ and plays a pivotal role in the biomineralization process of calcifying organisms [53]. Exposure to high *p*CO_2_ has been found to significantly hinder the expression of CA transcripts and decrease CA activity in various mollusks [54,55,56]. The present study demonstrated a similar decrease in CA transcripts (CA2, CA3, CA14, αCA, and βCA), suggesting the presence of a conserved mechanism in the regulation of CA in the adaptive responses of marine calcifiers to OA. *DE-cadherin*, *perlucin 5*, and *protocadherin Fat 1* involved in biomineralization were downregulated in *A. japonicus*. Cadherin has been established as a crucial component of Wnt signaling, playing a role in the biomineralization process of pearl oysters, *Pincatada fucata* [57]. Perlucin is a water-soluble nonacidic protein with a calcium-dependent carbohydrate binding domain, facilitating the nucleation and growth of CaCO_3_ crystals [58].

Furthermore, low-pH stress induced a reduction in the transcriptional changes of crucial regulatory genes associated with larval skeletogenesis in echinoderms, such as C-type lectin (*clet*), vascular endothelial growth factor receptor (*vegfR*), and *ALX* homeobox 1 (*alx1*) [59]. Ion transporters play a vital role in biological mineralization by facilitating the transportation of calcium, carbonate, or bicarbonate to the mineralization site. The present study revealed that several genes involved in calcium transport or signaling were negatively affected, including *VWFA and cache domain-containing protein 1* (*CACHD1*) and *sodium/potassium/calcium exchanger*. *CACHD1* is known to play a significant role in the regulation of voltage-gated calcium channels, and a sodium/potassium/calcium exchanger can modulate the transport of Ca^2+^ in and out of the mantle cells of *Crassostrea gigas*, which could potentially be important ion suppliers for the biomineralization process [60,61].

The risk of extracellular acid–base imbalance is heightened by acidification stress, resulting in a decrease in the expression of genes involved in maintaining pH homeostasis, such as the proton channel, H(^+^)Cl(^−^) exchange transporter (*CLCN7*), and organic cation transporter (*Orct*). These findings collectively suggest that biomineralization serves as a distinct and specific indicator of OA stress during the doliolaria stage of *A. japonicus*, and the above biomineralization-related DEGs can be utilized for further investigation into the biomineralization mechanisms by which sea cucumbers exhibit resilience to OA.

#### 4.1.2. Warming Influenced Embryos and Larvae at the Molecular Level

Temperature is an essential factor influencing changes in gene expression during the early larval development of sea cucumbers, especially at the blastula stage, with the largest number of DEGs and significant developmental changes. In accordance with the aforementioned developmental promotion effects, gene profiling analysis revealed that blastula cultivated in warmer temperature environments exhibited gene expression patterns associated with the development and regulation of the cell cycle, such as the *cyclin* and *Wnt* families. *Wnt* genes encode secreted glycoproteins that serve as crucial signaling molecules in the regulation of cell proliferation, migration, and differentiation during the initial polarization of embryos and the specification of endomesoderm in various organisms [62,63]. The upregulation of four *Wnt* genes in OW treatments suggests that Wnt signaling is initiated earlier during the blastula stage, leading to accelerated development. Conversely, the downregulation of *cyclin* genes involved in cell division may serve as an energy conservation strategy in response to increased temperature.

In marine invertebrates, disruptions in cellular homeostasis often result in a significant transcriptional upregulation of genes associated with cellular stress responses [47,64,65]. Consequently, it is not surprising that *A. japonicus* exhibited a noticeable stress response at the transcript level under higher temperatures. The heat shock protein (Hsp) is a highly responsive multigene family that is particularly sensitive to diverse environmental stressors, and it plays vital roles in protein refolding and degradation. Various *Hsps* genes (*Hsp26*, *Hsp70*, and *Hsp90*) have been shown to increase gene expression across four larval stages of *A. japonicas* during heat stress. Consistent with the results of the present study, increased *Hsps* expression was observed in the sea cucumbers *Holothuria leucospilota* and *A. japonicus* under thermal stress [35,64]. Heat stress frequently results in an overabundance of ROS [66], necessitating the essential role of antioxidant enzymes in preserving cellular redox homeostasis in sea cucumbers, including superoxide dismutase, glutathione S-transferase, scavenger receptor cysteine-rich domain superfamily protein, and glutathione peroxidase. The dysregulation of numerous immune response genes, such as the complement system, *cathepsin*, *fucolectin*, *Lectin*, and *TNF receptor-associated factors*, primarily during the blastula stage, indicates that sea cucumbers exposed to elevated temperatures experience compromised immune functionality, rendering them highly susceptible to environmental pathogen infections.

#### 4.1.3. Molecular Response to Dual Stresses of OA and OW

When larval sea cucumbers were subjected to elevated temperature or increased *p*CO_2_, upregulation of genes related to stress response and antioxidant defense was observed to varying extents. However, under the stress of OAW, these genes were expressed in greater quantities and on a larger scale, indicating a higher overall energy input for cellular homeostasis regulation under dual stress than under one single stress. Another study on American lobster post-larvae (*Homarus americanus*) confirmed that the combined stress of elevated *p*CO_2_ and temperature has a more pronounced effect on gene expression regulation than the presence of a single stressor [67]. Furthermore, a greater number of immune-defense- and disease-related pathways (e.g., phagosome, antigen processing and presentation, pathogenic *Escherichia coli* infection, microRNAs in cancer, basal cell carcinoma, and pathways in cancer) were found to be enriched under dual stresses of high temperature and *p*CO_2_. This finding suggests that exposure to these dual stresses may lead to disorders in immune system function, thus increasing the susceptibility to various infections. Indeed, the combined effect of high temperature and *p*CO_2_ on various marine species was observed to induce transcriptional changes in genes associated with the immune system, resulting in dysregulation [67,68,69,70]. However, the transcriptional changes in *A. japonicus* in response to the combined effect of heat and acidification contradict the developmental results mentioned in the above studies. The observed transcriptional changes in larval *A. japonicus* exposed to the combined stress of heat and acidification appear to be different. However, as explained by Niemisto et al. [68] during the early stages of organism development, compensatory mechanisms may mask responses in other measured physiological, behavioral, or morphometric endpoints [68]. Therefore, despite the absence of significant differences in developmental performance between the larval individuals exposed to both stresses and the control group, the stress response appears to be front-loaded at the transcriptional level. Moreover, the transcriptional front-loading of the stress response toolkit in *A. japonicus* larvae indicates a prioritization of energy allocation towards the maintenance of cellular homeostasis under stresses of heat and acidification, resulting in reduced immune resistance and heightened system instability.

## 5. Conclusions

In summary, OW has been found to promote the embryo–larval development of a less-calcified echinoderm *A. japonicus*, and OA inhibited the developmental rate and body size, but OAW exhibited an antagonistic effect. Furthermore, large-scale gene expression profiling of *A. japonicus* across four crucial embryo–larval stages by RNA-seq technique has identified a cluster of key DEGs and KEGG pathways that are involved in the processes of stress response, biomineralization, and cellular homeostasis under exposure to OA and/or OW. These findings pertaining to the molecular responses of early stages of the sea cucumber *A. japonicus* contribute to the comprehension of the physiological mechanisms underlying the tolerance of CO_2_-driven seawater acidification and warming.

## Figures and Tables

**Table 1:** Parameters of carbonate chemistry in the seawater acidification and warming system.

Treatments	Measured			Calculated			
	Salinity	Temperature (°C)	pH	TA (mol/kg^−1^SW)	*p*CO_2_ (µatm)	Ω_ca_	Ω_ar_
CON	36.4 ± 0.2	19.1 ± 0.3	8.08 ± 0.07	2657 ± 54	602 ± 12	4.26 ± 0.64	2.77 ± 0.42
OA	36.4 ± 0.2	19.0 ± 0.4	7.74 ± 0.11	2667 ± 53	1471 ± 393	2.17 ± 0.46	1.41 ± 0.30
OW	36.4 ± 0.2	22.3 ± 0.3	8.12 ± 0.04	2573 ± 131	519 ± 65	4.86 ± 0.50	3.18 ± 0.32
OAW	36.4 ± 0.2	22.1 ± 0.2	7.72 ± 0.06	2619 ± 33	1507 ± 233	2.23 ± 0.30	1.41 ± 0.20

Seawater pH, salinity, and temperature (*n* = 60) in aquariums were monitored daily; total alkalinity (TA) was measured on a 3-day basis (all *n* = 20). *p*CO_2_ and calcite and aragonite saturation states were calculated using CO2SYS software. Values are expressed as means ± SD. CON: 19 °C, 400 μatm; OA: 19 °C, 1000 μatm; OW: 22 °C, 400 μatm; OWA: 22 °C, 1000 μatm.

**Table 2:** Key DEGs identified at different stages under different treatments.

Treatments	OA vs. CON				OW vs. CON				OAW vs. CON			
Developmental Stage	Blastula	Gastrula	Auricularia	Doliolaria	Blastula	Gastrula	Auricularia	Doliolaria	Blastula	Gastrula	Auricularia	Doliolaria
NR Description	log_2_(FC)	log_2_(FC)	log_2_(FC)	log_2_(FC)	log_2_(FC)	log_2_(FC)	log_2_(FC)	log_2_(FC)	log_2_(FC)	log_2_(FC)	log_2_(FC)	log_2_(FC)
Development and regulation of cell cycle												
cyclin B	−2.24 ↓	-	-	−3.99 ↓	−5.98 ↓	-	-	-	-	-	-	-
cell division cycle protein 20	1.70 ↑	-	-	−1.65 ↓	−6.36 ↓	-	-	-	2.02 ↑	−0.41 ↓	-	-
cyclin A	-	-	-	-	−4.51 ↓	-	-	-	−1.03 ↓	-	-	-
cyclin E	−1.10 ↓	-	-	−5.49 ↓	−5.13 ↓	-	-	-	-	-	-	-
putative cyclin-G1	−1.35 ↓	1.04 ↑	-	-	−4.31 ↓	-	-	-	−1.13 ↓	-	-	-
putative cyclin-J	−1.43 ↓	-	-	-	−5.24 ↓	-	-	-	−1.51 ↓	-	-	-
Wnt16	-	-	-	-	5.45 ↑	-	-	-	-	-	-	-
Wnt6	1.35 ↑	-	-	−6.13 ↓	2.42 ↑	0.85 ↑	-	-	1.12 ↑	1.75 ↑	-	-
Wnt4	-	-	-	−6.13 ↓	5.46 ↑	-	-	-	-	2.86 ↑	1.99 ↑	-
Wnt3	-	1.20 ↑	-	−2.63 ↓	0.87 ↑		-	-	1.76 ↑	-	-	-
Immune response and antioxidant defense												
heat shock protein 26	-	-	−3.77 ↓	1.32 ↑	5.48 ↑	4.54 ↑	5.02 ↑	1.65 ↑	-	−1.21 ↓	−1.78 ↓	-
heat shock protein 70	-	-	-	3.87 ↑	3.07 ↑	3.46 ↑	8.51 ↑	5.32 ↑	5.73 ↑	6.31 ↑	-	6.94 ↑
heat shock protein 90	-	-	-	6.52 ↑	2.57 ↑	1.49 ↑	-	4.32 ↑	1.05 ↑	1.13 ↑	5.43 ↑	9.95 ↑
superoxide dismutase	-	1.33 ↑	-	8.48 ↑	6.23 ↑	-	-	6.98 ↑	-	1.25 ↑	-	7.00 ↑
glutathione S-transferase	1.48 ↑	1.21 ↑	-	-	8.19 ↑	1.18 ↑	−1.24 ↓	6.86 ↑	1.64 ↑	1.51 ↑	−1.45 ↓	8.89 ↑
scavenger receptor cysteine-rich domain superfamily protein	1.99 ↑	1.70 ↑	-	-	7.58 ↑	-	1.65 ↑	-	1.18 ↑	1.16 ↑	-	-
glutathione peroxidase	2.09 ↑	-	-	7.40 ↑	4.89 ↑	1.42 ↑	-	7.14 ↑	7.39 ↑	3.31 ↑	-	-
fucolectin	-	-	-	1.00 ↑	12.26 ↑	-	-	-	-	-	-	-
putative IgGFc-binding protein	-	-	-	−1.93 ↓	5.18 ↑	-	-	-	3.06 ↑	3.59 ↑	-	-
cathepsin	4.70 ↑	1.07 ↑	-	8.64 ↑	9.76 ↑	1.00 ↑	−1.58 ↓	8.27 ↑	1.83 ↑	1.13 ↑	−1.34 ↓	7.48 ↑
complement component 3-2	2.54 ↑	-	-	−2.08 ↓	2.44 ↑	-	-	-	2.48 ↑	1.79 ↑	-	-
complement factor B	-	-	-	−2.05 ↓	4.13 ↑	-	-	-	-	1.52 ↑	-	-
putative complement factor H	-	-	-	5.85 ↑	8.24 ↑	-	-	-	-	-	-	-
Lectin 1	-	-	-	−3.77 ↓	−1.22 ↓	-	4.51 ↑	-	-	-	3.76 ↑	-
TNF receptor-associated factor 2	-	-	-	-	−5.61 ↓	-	-	-	-	-	-	-
TNF receptor-associated factor 6	−3.04 ↓	-	-	−2.82 ↓	−9.35 ↓	-	-	-	−2.09 ↓	1.23 ↑	-	-
LPS-induced TNF-alpha factor	-	1.05 ↑	-	1.08 ↑	7.57 ↑	-	-	-	-	1.07 ↑	-	-
Biomineralization and Osteoblast												
putative transcription factor SOX-4	-	-	-	-	−2.74 ↓	-	-	-	1.60 ↑	-	-	-
ets1..2 transcription factor	1.56 ↑	-	-	−2.01 ↓	−2.79 ↓	-	-	-	1.94 ↑	-	-	-
fox-1 homolog 1-like isoform X9	-	1.66 ↑	-	−5.14 ↓	-	-	-	-	-	-	-	-
alx1	-	1.35 ↑	-	−7.15 ↓	−4.86 ↓	-	-	-	-	-	-	-
transcription factor SOX-21	1.635 ↑	1.34 ↑	-	−4.54 ↓	−1.57 ↓	-	-	-	-	-	-	-
T-box transcription factor	-	-	-	−2.05 ↓	6.37 ↑	-	-	-	1.35 ↑	3.55 ↑	-	-
fibroblast growth factor receptor	−1.77 ↓	1.43 ↑	-	−2.66 ↓	−5.19 ↓	1.53 ↑	-	-	1.90 ↑	4.39 ↑	-	-
TGFB-induced factor homeobox 1	1.70 ↑	-	-	−3.88 ↓	−1.94 ↓	-	-	-	2.09 ↑	-	-	-
vascular endothelial growth factor receptor 2-like isoform X4	-	-	-	−6.14 ↓	-	-	-	-	-	-	-	-
putative vascular endothelial growth factor receptor 1	−1.56 ↓	1.49 ↑	-	−1.65 ↓	−5.23 ↓	1.13 ↑	-	-	−1.16 ↓	1.52 ↑	-	-
GSK-3-binding protein	-	-	-	−5.04 ↓	-	-	-	-	-	-	-	-
putative C-type lectin domain family 19 member A	-	-	-	−6.02 ↓	10.13 ↑	-	−2.11 ↓	-	-	5.69 ↑	-	-
C-type lectin domain-containing protein	-	-	-	−4.76 ↓	-	-	-	-	-	-	-	-
cyclophilin	5.84 ↑	-	-	7.91 ↑	-	-	-	6.60 ↑	-	-	-	8.57 ↑
3 alpha procollagen precursor	-	-	-	−2.06 ↓	2.01 ↑	1.45 ↑	-	-	-	2.83 ↑	-	-
carbonic anhydrase 14	-	-	-	−4.73 ↓	3.31 ↑	-	−1.50 ↓	-	-	1.32 ↑	-	-
carbonic anhydrase 3	-	-	-	−2.27 ↓	6.4815 ↑	-	-	-	-	2.13 ↑	-	-
carbonic anhydrase 2	−1.76 ↓	-	-	−3.54 ↓	4.6567 ↑	-	-	-	−1.26 ↓	1.92 ↑	-	−8.86 ↓
alpha-carbonic anhydrase	-	-	-	−5.19 ↓	-	-	-	-	-	-	-	-
beta-carbonic anhydrase	-	-	-	−3.33 ↓	-	-	-	-	-	-	-	-
protocadherin fat 1-like isoform X5	-	1.76 ↑	-	−2.38 ↓	−2.58 ↓	1.09 ↑	-	-	2.47 ↑	1.41 ↑	-	-
DE-cadherin	-	-	-	−4.19 ↓	-	-	-	-	-	-	-	-
serine threonine-protein ki-se mTOR	-	1.24 ↑	-	−5.90 ↓	−1.23 ↓	-	-	-	-	1.54 ↑	-	-
vacuolar protein sorting-associated protein 13C	-	-	-	−2.75 ↓	−2.98 ↓	-	-	-	−1.64 ↓	4.56 ↑	-	-
perlucin 5	-	-	-	−3.23 ↓	-	-	-	-	-	-	-	-
Transport of calcium ions and maintenance of ion homeostasis												
VWFA and cache domain-containing protein 1-like	-	1.70 ↑	-	−4.76 ↓	−2.24 ↓	1.46 ↑	-	-	2.24 ↑	1.82 ↑	-	-
sodium/potassium/calcium exchanger 3 isoform X1	−2.31 ↓	-	-	−6.38 ↓	−1.62 ↓	-	1.84 ↑	-	−1.69 ↓	2.57 ↑	-	-
probable sodium/potassium/calcium exchanger CG1090	-	-	-	−6.57 ↓	-	-	-	-	-	-	-	-
proton channel	-	1.37 ↑	-	−7.57 ↓	−4.56 ↓	1.47 ↑	-	-	-	2.78 ↑	-	-
H^+^Cl^−^ exchange transporter 7	-	1.54 ↑	-	−5.04 ↓	−3.62 ↓	-	-	-	-	2.45 ↑	-	-
chloride channel protein	−1.62 ↓	1.2076 ↑	-	−3.49 ↓	−2.96 ↓	1.10 ↑	-	-	−1.38 ↓	1.74 ↑	-	-
caveolin	-	-	-	1.43 ↑	7.51 ↑	-	-	-	−1.63 ↓	4.12 ↑	-	-
organic cation transporter	2.42 ↑	-	-	−4.86 ↓	10.53 ↑	1.51 ↑	1.51 ↑	-	2.35 ↑	1.89 ↑	-	-
calmodulin	5.38 ↑	-	-	11.45 ↑	−1.93 ↓	4.11 ↑	4.11 ↑	5.64 ↑	-	5.77 ↑	6.73 ↑	−3.82 ↓
pct-1	-	-	-	-	2.75 ↑	-	-	-	-	-	-	-
transmembrane protein 120A	-	-	-	-	−3.04 ↓	-	-	-	-	-	-	-

Note: FC represents change fold, ↑ marks upregulated DEGs, and ↓ marks downregulated DEGs. CON: 19 °C, 400 μatm; OA: 19 °C, 1000 μatm; OW: 22 °C, 400 μatm; OWA: 22 °C, 1000 μatm.

## Data Availability

The raw datasets generated and/or analyzed during the current study are available from the corresponding author upon reasonable request.

## Supplementary Materials

The following supporting information can be downloaded at: https://www.mdpi.com/article/10.3390/biology12121520/s1, Figure S1: An experimental design schematic; Figure S2: Veen plot of DEGs; Table S1: Primers used in the study; Table S2: Summary of transcriptomic sequencing data.
