# Supplementary material for: Transcriptomic Responses of a Lightly Calcified Echinoderm to Experimental Seawater Acidification and Warming during Early Development
Source: Biology (Basel). 2023 Dec 13;12(12):1520. doi: 10.3390/biology12121520 (PMC10740944; doi:10.3390/biology12121520)
Supplement: Supplementary file 1 [file biology-12-01520-s001.zip › Figure S2.pdf]

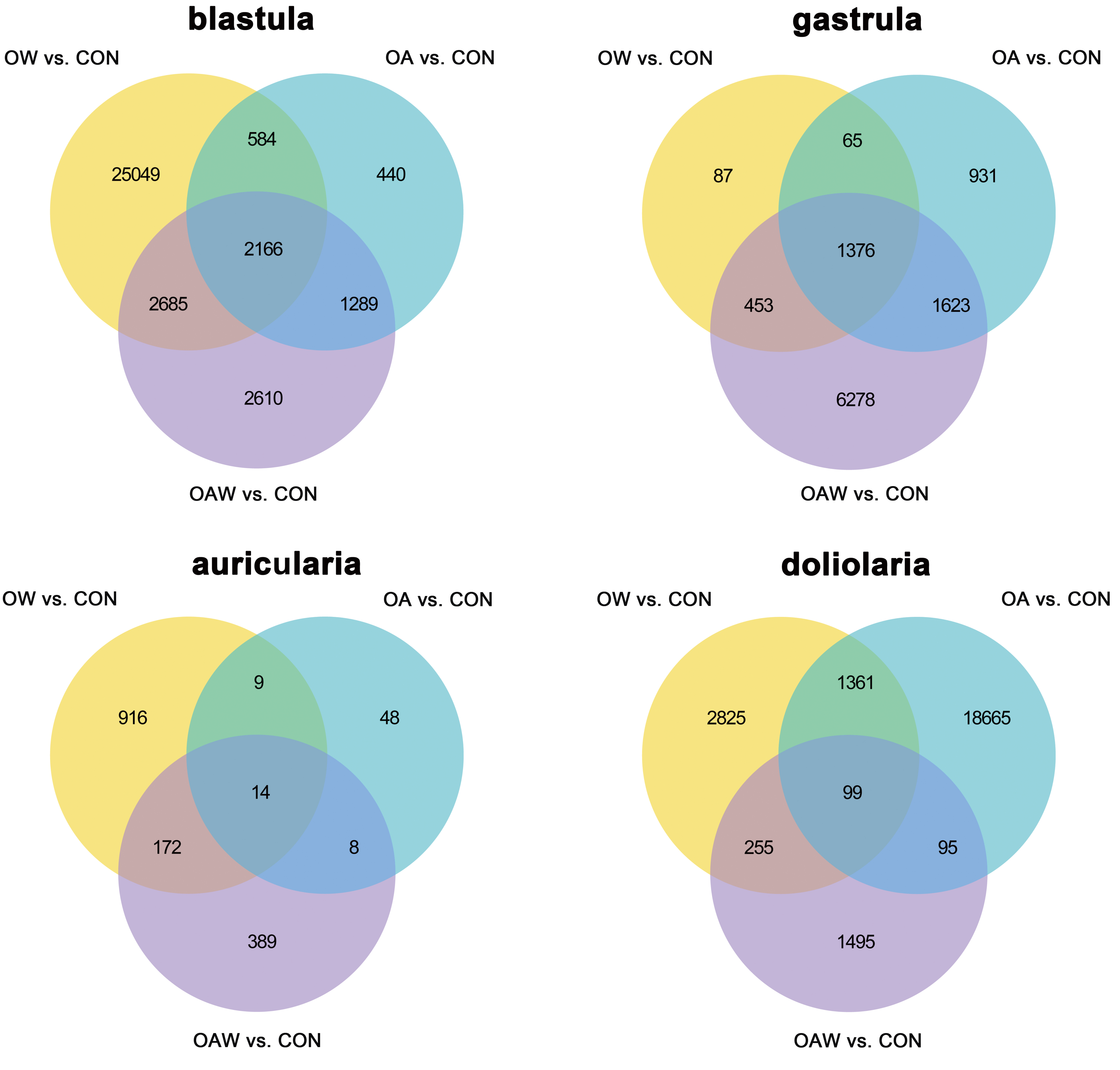
blastula
OW vs. CON
OA vs. CON
OAW vs. CON
25049
584
440
2166
2685
1289
2610
gastrula
OW vs. CON
OA vs. CON
OAW vs. CON
87
65
931
1376
453
1623
6278
auricularia
OW vs. CON
OA vs. CON
OAW vs. CON
916
9
48
14
172
8
389
doliolaria
OW vs. CON
OA vs. CON
OAW vs. CON
2825
1361
18665
99
255
95
1495
