# Supplementary material for: Transcriptomic Responses of a Lightly Calcified Echinoderm to Experimental Seawater Acidification and Warming during Early Development
Source: Biology (Basel). 2023 Dec 13;12(12):1520. doi: 10.3390/biology12121520 (PMC10740944; doi:10.3390/biology12121520)
Supplement: Supplementary file 1 [file biology-12-01520-s001.zip › Tables S1 and S2.pdf]

Table S1. Primers used in the study

Gene	Annealing temperature	Primer sequence	
		**Sense 5'-3'**	**Antisense 5'-3'**
cytochrome P450 2J6	55˚C	CTTGAACGATTGCAGGAGGG	GTTGGATACCTTTCGCGTCC
G-protein signaling modulator	56˚C	TTGAGATGCTAATGCGATGC	TCGGTTCTCTGCTCTTCGAT
collagen	61˚C	CAGGTGTCCAGGGACCTAAA	AGCGCCAGTAACTCCAGTGT
heat shock protein 90	61˚C	CCAGTTTGGTGTTGGCTTTT	CTGGCTGTGTTTCTTGACGA
NADH dehydrogenase	55˚C	CTCGTAATCGCCGTTATGGT	TATCCATTGGGTGGGAAGAA
Bone morphogenetic protein 2	61˚C	CGTCGTGACAAAAGGGAAAT	TGTAATAAGCGCGTGAGTCG
Tgif	55˚C	GCTCGCTCTCAAAAGGACAAG	GACACCATCACCACCAGCA
Wnt 8	55˚C	CAGATGAACAGCAAACCACCA	GGGAGCGGTGCGATAGTAAG

Table S2. Summary of transcriptomic sequencing data

Stage	Treatment	Sample	Raw Data	Clean Data	Valid Ratio (%)	Mapped Reads	Mapped Ratio (%)	Q20(%)	Q30 (%)	GC Content%
Blastula	CON	B1_A1	68,216,408	66,865,914	98.02	49,920,118	74.66	98.36	94.9	39.53
		B1_A2	58,354,486	57,307,170	98.21	42,904,656	74.87	98.32	94.79	40.11
		B1_A3	51,733,248	50,730,226	98.06	38,178,462	75.26	98.38	94.95	40.15
	OA	B1_C1	53,997,880	53,028,028	98.20	40,221,494	75.85	98.42	94.88	38.84
		B1_C2	53,959,424	52,989,766	98.20	40,966,230	77.31	98.49	95.2	40.76
		B1_C3	56,311,882	55,194,104	98.02	42,760,568	77.47	98.51	95.25	41.05
	OW	B1_B1	54,658,734	53,679,476	98.21	40,589,590	75.61	98.38	94.88	39.66
		B1_B2	68,785,244	65,708,830	95.53	49,094,930	74.72	98.39	94.92	38.81
		B1_B3	55,752,068	53,694,044	96.31	41,377,084	77.06	98.48	95.18	41.05
	OAW	B1_D1	63,280,640	62,142,800	98.20	47,733,902	76.81	98.44	95.04	40.76
		B1_D2	59,186,792	58,150,330	98.25	44,108,498	75.85	98.44	95.07	39.93
		B1_D3	58,147,718	56,903,742	97.86	44,139,780	77.57	98.52	95.31	41.09
Gastrula	CON	G2_A1	55,324,616	53,230,134	96.21	40,640,070	76.35	98.41	95.01	39.56
		G2_A2	57,838,010	56,516,334	97.71	44,164,930	78.15	98.44	95.06	41.53
		G2_A3	54,855,936	53,857,586	98.18	41,054,650	76.23	98.28	94.66	39.99
	OA	G2_C1	54,816,816	53,437,148	97.48	41,824,920	78.27	98.49	95.22	41.08
		G2_C2	65,259,354	63,123,980	96.73	50,690,016	80.30	98.64	95.63	43.42
		G2_C3	54,822,384	54,029,356	98.55	42,022,200	77.78	98.44	95.09	41.62
	OW	G2_B1	59,578,780	58,416,556	98.05	45,064,050	77.14	98.42	95	41.01
		G2_B2	50,672,768	49,653,458	97.99	38,397,464	77.33	98.36	94.86	40.8
		G2_B3	53,466,492	52632,858	98.44	40,618,396	77.17	98.42	95.01	40.9

	OAW	G2_D1	54,918,784	53,653,396	97.70	42,350,944	78.93	98.55	95.35	42.56
		G2_D2	58,047,184	57,100,486	98.37	44,169,230	77.35	98.47	95.15	41.38
		G2_D3	64,108,428	63,138,568	98.49	48,807,650	77.30	98.5	95.21	41.29
**Auricularia**	CON	A3_A1	49,143,600	48,287,936	98.26	36,397,308	75.38	98.46	95.16	40.08
		A3_A2	66,288,238	65,036,376	98.11	48,918,260	75.22	98.46	95.15	40.68
		A3_A3	46,878,742	46,045,986	98.22	34,204,830	74.28	98.41	95	39.23
	OA	A3_C1	51,203,618	49,528,940	96.73	38,947,808	78.64	98.53	95.3	42.14
		A3_C2	62,329,326	60,513,956	97.09	46,990,706	77.65	98.4	95.03	40.86
		A3_C3	42,798,728	41,464,062	96.88	31,550,048	76.09	98.25	94.64	39.27
	OW	A3_B1	63,549,768	62,062,396	97.66	46,846,594	75.48	98.37	94.9	40.26
		A3_B2	52,039,874	51,068,308	98.13	38,125,134	74.66	97.89	93.59	39.77
		A3_B3	57,143,634	56,277,324	98.48	42,446,326	75.42	98.41	95.01	39.96
	OAW	A3_D1	58,644,454	57,518,446	98.08	43,498,160	75.62	98.38	94.93	39.21
		A3_D2	41,837,242	40,906,436	97.78	30,554,620	74.69	98.16	94.3	38.33
		A3_D3	58,978,404	57,877,840	98.13	44,458,532	76.81	98.4	95.04	40.84
**Doliolaria**	CON	D4_A1	54,767,706	53,810,376	98.25	41,562,576	77.24	98.47	95.22	41.13
		D4_A2	68,970,942	67,613,596	98.03	52,447,866	77.57	98.39	95.05	41.29
		D4_A3	53,647,426	52,462,220	97.79	41,390,438	78.90	98.42	95.04	43.27
	OA	D4_C1	43,742,834	42,258,498	96.61	32,279,970	76.39	98.41	95.08	40.06
		D4_C2	54,092,350	52,968,858	97.92	40,936,416	77.28	98.46	95.12	40.86
		D4_C3	47,738,822	46,711,608	97.85	36,631,158	78.42	98.67	95.7	42.61
	OW	D4_B1	57,414,410	55,729,266	97.06	41,640,154	74.72	98.37	95.1	40.43
		D4_B2	57476240	55,969,908	97.38	42,419,064	75.79	98.39	95.03	41.03
		D4_B3	76820576	75,105,010	97.77	57,219,522	76.19	98.33	94.91	40
	OAW	D4_D4	65,533,786	64,319,512	98.15	49,804,042	77.43	98.4	94.94	42.1

	D4_D2	57,393,394	56,336,964	98.16	44,097,202	78.27	98.42	94.95	42.02
	D4_D3	53,069,730	52,187,932	98.34	40,384,348	77.38	98.47	95.17	41.36

Note: Q20, base sequencing error probability <1%; Q30, base sequencing error probability <0.1%. CON: 19˚C, 400 μatm; OA: 19˚C, 1000 μatm; OW: 22˚C, 400 μatm; OWA: 22˚C, 1000 μatm.
