# Supplementary figures and images for: Transcriptomic Responses of a Lightly Calcified Echinoderm to Experimental Seawater Acidification and Warming during Early Development
Source: Biology (Basel). 2023 Dec 13;12(12):1520. doi: 10.3390/biology12121520 (PMC10740944; doi:10.3390/biology12121520)

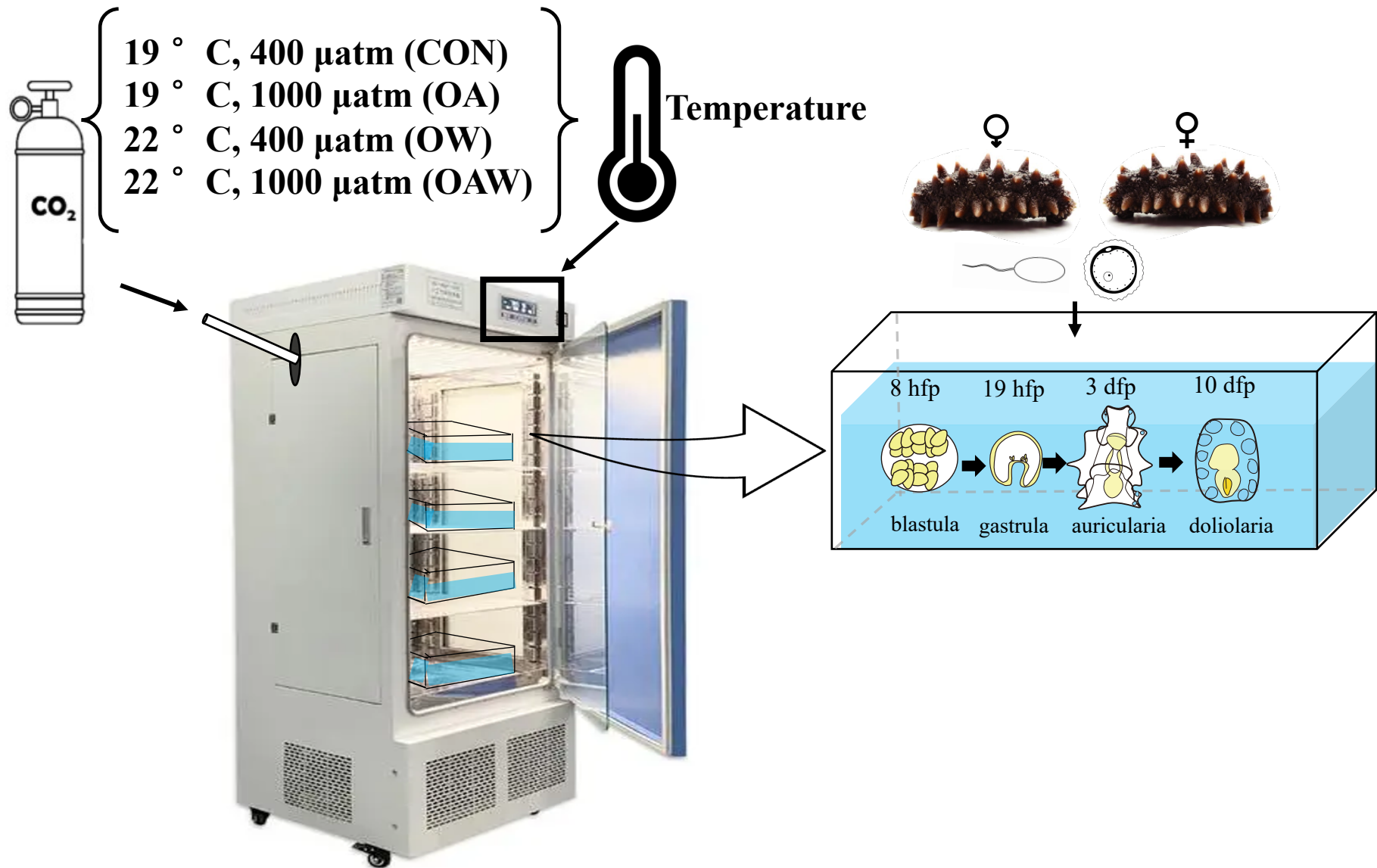

Supplement: Supplementary file 1 [file biology-12-01520-s001.zip › Figure S1.pdf]
